# Suppression of SNARE‐dependent exocytosis in retinal glial cells and its effect on ischemia‐induced neurodegeneration

**DOI:** 10.1002/glia.23144

**Published:** 2017-04-03

**Authors:** Lysann Wagner, Thomas Pannicke, Vanessa Rupprecht, Ina Frommherz, Cornelia Volz, Peter Illes, Johannes Hirrlinger, Herbert Jägle, Veronica Egger, Philip G. Haydon, Frank W. Pfrieger, Antje Grosche

**Affiliations:** ^1^ Paul Flechsig Institute of Brain Research, University of LeipzigLiebigstr. 19Leipzig04103Germany; ^2^ Institute of Zoology, University of RegensburgUniversitätsstr. 31Regensburg93040Germany; ^3^ Department of OphthalmologyUniversity of RegensburgFranz‐Josef‐Strauß‐Allee 1Regensburg93953Germany; ^4^ Rudolf Boehm Institute for Pharmacology and Toxicology, University of Leipzig, Härtelstr. 16/18, 04107LeipzigGermany; ^5^ Carl Ludwig Institute of Physiology, University of LeipzigLiebigstr. 27Leipzig04103Germany; ^6^ Department of NeurogeneticsMax‐Planck‐Institute for Experimental MedicineHermann‐Rein‐Str. 3Göttingen37075Germany; ^7^ Department of NeuroscienceTufts University School of Medicine136 Harrison AvenueBostonMassachusetts02111USA; ^8^ Institute of Cellular and Integrative Neurosciences, CNRS UPR 3212, University of Strasbourg5 rue Blaise PascalStrasbourg Cedex67084France; ^9^ Institute of Human Genetics, University of RegensburgFranz‐Josef‐Strauß‐Allee 1Regensburg93953Germany

**Keywords:** glutamate, gliotransmitter, ischemia, Müller cell, retina

## Abstract

Nervous tissue is characterized by a tight structural association between glial cells and neurons. It is well known that glial cells support neuronal functions, but their role under pathologic conditions is less well understood. Here, we addressed this question in vivo using an experimental model of retinal ischemia and transgenic mice for glia‐specific inhibition of soluble *N*‐ethylmaleimide‐sensitive factor attachment protein receptor (SNARE)‐dependent exocytosis. Transgene expression reduced glutamate, but not ATP release from single Müller cells, impaired glial volume regulation under normal conditions and reduced neuronal dysfunction and death in the inner retina during the early stages of ischemia. Our study reveals that the SNARE‐dependent exocytosis in glial cells contributes to neurotoxicity during ischemia in vivo and suggests glial exocytosis as a target for therapeutic approaches.

## 1 INTRODUCTION

Neuroglial cells such as astrocytes and retinal Müller cells not only fill the space between neurons but they represent essential partners. Under normal conditions, they support neuronal function in different ways including potassium buffering, transmitter uptake and the provision of energy substrates (Bringmann et al., 2006; Parpura et al., 2012). Their role under pathologic conditions caused by mutations, ischemia or injuries is less well understood, they may exacerbate or mitigate neuronal damage. Extracellular accumulation of glutamate, the major neurotransmitter in the CNS, damages neurons in different pathologies including ischemia (Brassai, Suvanjeiev, Bán, & Lakatos, 2015; Osborne et al., 2004), but the contribution of glial cells remains unclear (Rossi & Volterra, 2009). Under normal conditions, glial cells control the extracellular glutamate concentration by electrogenic glutamate transporters (Bringmann et al., 2013; Grewer & Rauen, 2005; Rauen, 2000). Consequently, an impairment of glial glutamate uptake for example due to accumulation of extracellular potassium (Billups & Attwell, 1996; Maguire, Simko, Weinreb, & Ayoub, 1998; Rossi, Oshima, & Attwell, 2000; Szatkowski, Barbour, & Attwell, 1990) could cause loss of neurons (Rossi, Brady, & Mohr, 2007).

Studies within the last decade have shown that glial cells can release transmitters (which are called gliotransmitters) in a calcium‐ and soluble *N*‐ethylmaleimide‐sensitive factor attachment protein receptor (SNARE)‐dependent manner (Marchaland et al., 2008; Martineau, 2013; Montana, Malarkey, Verderio, Matteoli, & Parpura, 2006; Slezak et al., 2012). The physiologic relevance of this process is debated: it may contribute to glial volume regulation (Slezak et al., 2012) and modulate neuronal activity (Araque et al., 2014; Chen et al., 2012; Perez‐Alvarez, Navarrete, Covelo, Martin, & Araque, 2014; Takata et al., 2011). Here, we studied the contribution of glial SNARE‐dependent exocytosis to neurodegeneration in the retina. To this end, we used an established model of transient ischemia that triggers robust neuronal dysfunction and degeneration (Osborne et al., 2004; Pannicke et al., 2014). To interfere with glial exocytosis, we used a mouse line enabling inducible expression of a dominant‐negative domain of synaptobrevin (dnSNARE mice; Pascual et al., 2005). Our results reveal that glia‐specific inhibition of SNARE‐dependent signaling protects retinal neurons from dysfunction and degeneration.

## 2 MATERIALS AND METHODS

### 2.1 Materials

All substances were purchased from Sigma‐Aldrich (Taufkirchen, Germany) unless stated otherwise. Papain was obtained from Roche (Mannheim, Germany). Chloromethyl‐tetramethyl‐rosamine (Mitotracker Orange), NP‐EGTA (o‐nitrophenyl EGTA), NPE‐ATP (P(3)‐[1‐(2‐nitrophenyl)]ethyl ester of ATP), and Fluo‐4 AM were from Molecular Probes (Life Technologies, Carlsbad, CA). For immunocyto‐ and histochemical staining, the following primary antibodies were used: rabbit anti‐Kir4.1 (1:200; Sigma‐Aldrich), mouse anti‐glial fibrillary acidic protein (GFAP; 1:200; G‐A‐5 clone, Sigma‐Aldrich), goat anti‐calretinin (1:500, Swant, Marly, Switzerland), mouse anti‐calbindin (1:400, Swant), rabbit anti‐PKCα (1:300, Santa Cruz Biotechnology, Heidelberg, Germany), rabbit anti‐cellular retinaldehyde‐binding protein (CRALBP; 1:300, Santa Cruz), rabbit‐anti‐Iba1 (1:500, Wako Chemicals, Neuss, Germany), and mouse anti‐glutamine synthetase (1:1000, Merck Millipore, Darmstadt, Germany). As secondary antibodies, we used Cy5‐conjugated donkey anti‐goat, Cy3‐conjugated donkey anti‐rabbit, Cy2‐conjugated donkey anti‐mouse, Cy3‐conjugated goat anti‐rabbit, and Cy2‐conjugated goat anti‐mouse. All secondary antibodies were obtained from Dianova (Hamburg, Germany) and applied at 1:200 dilution. Apoptosis was detected by the in situ cell death detection kit (Roche, Mannheim, Germany).

### 2.2 Animals

All experiments were performed in accordance with the European Community Council Directive 2010/63/EU after approval by the local authorities (TVV 54/12 approved by the state directorate Leipzig). Animals were maintained with free access to water and food in an air‐conditioned room on a 12‐hr light‐dark cycle. Transgenic dnSNARE mice were bred as described earlier (Pascual et al., 2005). These mice use the Tet‐Off system to express a dominant negative subdomain of synaptobrevin (VAMP2) and enhanced green fluorescent protein (EGFP) under the control of a human GFAP promoter fragment. To prevent transgene expression during development, the animals received doxycycline until weaning (25 μg/ml in drinking water). Homozygous mice deficient in the nucleotide receptor P2Y_1_ (P2Y_1_R‐KO, 3–5 months old; Fabre et al., 1999) and wildtype littermates were genotyped according to Pannicke et al. (2014).

### 2.3 Measurement of transmitter release in single Müller cells

Transmitter release from acutely isolated Müller cells was measured using a fluorimetric enzyme assay as described (Slezak et al., 2012). Briefly, retinae were treated with papain (0.2 mg/ml; Roche Molecular Biochemicals) for 30 min at 37°C in the dark in Ca^2+^‐ and Mg^2+^‐free extracellular solution (140 mM NaCl, 3 mM KCl, 10 mM HEPES, 11 mM glucose, pH 7.4). Before detecting glutamate release, glutamine (0.25 mM) and glutamate (0.5 mM) were supplemented to maintain high intracellular glutamate levels. After several washes, retinae were triturated in extracellular solution (with 1 mM MgCl_2_ and 2 mM CaCl_2_) containing all components of the Amplex® Red Glutamic Acid kit, D,L‐threo‐beta‐benzyloxyaspartate (TBOA, 200 µM) to block glial glutamate uptake and NP‐EGTA (10 µM) or NPE‐ATP at varying concentrations. Subsequently, the cell suspension was mixed with 1.5% agarose and incubated for 30 min in the recording chamber at 37°C. At each recording session, we measured cells from one wild type mouse and one dnSNARE mouse using the same assay solution freshly prepared for the actual experiment. This rules out confounding effects of differences in the assay composition. If blockers were tested, they were co‐applied with the gel‐enzyme‐mixture and also preincubated for 30 min. The combination of enzymes generates H_2_O_2_ only in the presence of glutamate. H_2_O_2_ together with Amplex® Red serve as substrates for horseradish peroxidase to generate resorufin. Resorufin fluorescence was detected with a confocal laser microscope (LSM510 Meta, 100×/1.3 Plan‐Neofluar oil, Zeiss, Oberkochen, Germany; 543 nm helium‐neon laser, 585 nm long pass filter, pinhole maximally open). Images were taken from the endfeet of Müller cells, which were identified based on their unique morphology. To detect ATP release, glutamate‐specific enzymes (L‐glutamate oxidase, L‐glutamate–pyruvate transaminase), TBOA, and alanine were replaced by glycerol kinase (10 U/ml), glycerol‐3‐phosphate oxidase (10 U/ml) and glycerol (500 µM) from the Amplex® Red assay. The combination of enzymes and substrates generates H_2_O_2_ (and finally resorufin) only in the presence of ATP (Dale & Frenguelli, 2012).

To trigger calcium‐dependent release, intracellular calcium rises were induced by four UV‐pulses (351 nm/364 nm Enterprise UV Laser, 500 ms at maximal intensity) to uncage calcium from NP‐EGTA or to release ATP from NPE‐ATP. Peak amplitudes were calculated as difference between mean fluorescence intensity across four time points acquired before and after the UV pulses. To validate concentration‐dependent detection of glutamate or ATP, we performed the enzymatic assay under cell‐free conditions. To detect glutamate, extracellular solution with all enzymatic components of the Amplex® Red Glutamic Acid kit, TBOA (200 µM), and 1.5% agarose but without cells was filled into the recording chamber. Extracellular solution containing defined concentrations of glutamate was added to the recording chamber. Similarly, we validated the ATP detection assay using a similar procedure, but implementing glycerol kinase, glycerol‐3‐phosphate oxidase and glycerol. We recorded resorufin fluorescence under cell‐free conditions on a microplate reader (HTS7000 Bio Assay Reader Perkin‐Elmer, Rodgau Jügesheim, Germany).

### 2.4 Calcium measurements in single Müller cells

Cells were isolated as described for the measurements of transmitter release and incubated with Fluo4 AM (1 µM) and with or without NP‐EGTA and NPE‐ATP. ATP was applied by slow perfusion (600 µl/min) to induce calcium transients or by photolysis of NPE‐ATP as described for glutamate release experiments. The uncaging protocols are described in the figure legends (Figure 2).

### 2.5 Calcium measurements on retinal whole‐mounts

Retinae were prepared from male mice (P23–P90, C57BL/6) and transferred onto a net‐insert in a microdish filled with 1 ml artificial cerebrospinal fluid (ACSF) containing in mM: 125 NaCl, 26 NaHCO_3_, 1.25 NaH_2_PO_4_, 20 glucose, 2.5 KCl, 1 MgCl_2_, and 2 CaCl_2_. The solution was aerated with carbogen (95%O_2_, 5% CO_2_). Whole‐mounts were incubated in the dark for 1 hr at room temperature (22°C) with the fluorescent calcium indicator Fluo‐4 AM (15 µM, Invitrogen, Carlsbad, CA) added to the ACSF to selectively load Müller cells. VEGF (30 ng/ml) was bath‐applied in a closed perfusion circuit with a total volume of 14 ml. For calcium imaging the whole‐mount was transferred to a submerged chamber perfused with ACSF and held in place by a nylon grid with the ganglion cell layer (GCL) facing toward the water immersion objective (NA 1.0; Nikon Instruments, Tokyo, Japan). Retinal cells were visualized by gradient contrast using a Femto‐2D‐uncage microscope (Femtonics, Budapest, Hungary) controlled by MES v4.5.613 software (Femtonics). A tunable, Verdi‐pumped Ti:Sa laser (Chameleon Ultra, Coherent, Santa Clara, CA) was used for excitation of Fluo‐4 AM at 840 nm. Green fluorescence images were collected both in the epi‐ and transfluorescence mode. Müller cell endfeet (MCE) were imaged in frame scan mode for 2 min (rate: 20 Hz; size: 40 × 83 µm corresponding to ∼9 endfeet; image resolution: 1.6 µm per pixel. For the quantitative analysis, endfeet of Müller cells were marked as region of interest (ROI). Average or integral of pixels in these ROIs were averaged and changes in calcium were measured as ΔF/F and compared between different conditions. Control experiments included consecutive frame scans without drug application. Offline data analysis was performed using custom macros written in IGOR Pro (Wavemetrics, Lake Oswego, OR) and MES (Femtonics) and custom MATLAB (MathWorks, Ismaning, Germany) scripts.

### 2.6 Retinal ischemia

The high intraocular pressure (HIOP) method was used to induce transient ischemia in the retina as reported (Pannicke et al., 2014). Briefly, animals were anesthetized by intraperitoneal (ip) injections of ketamine (100 mg/kg body weight; Ratiopharm, Ulm, Germany), xylazine (5 mg/kg; Bayer Vital, Leverkusen, Germany), and atropine sulfate (100 mg/kg; Braun, Melsungen; Germany). The anterior chamber of one eye was cannulated from the pars plana by a 30‐gauge infusion needle connected to Deltajonin® (Deltaselect, Dreieich, Germany) bottle. The contralateral eye remained untreated and served as control. To interrupt retinal blood supply, the intraocular pressure was raised to 160 mm Hg for 90 min by elevating the bottle. Animals were sacrificed 14 hr, 1 or 7 days postoperation with carbon dioxide.

### 2.7 Volume regulation

Volume changes in retinal Müller cells were measured as described (Slezak et al., 2012). Briefly, retinal slices were loaded with the vital dye Mitotracker Orange (10 µM, excitation: 543 nm, emission: 560 nm long‐pass filter; Life Technologies), which is preferentially taken up by Müller cells (Uckermann et al., 2004). Slices were exposed to hypotonic solution (60% of control osmolarity using distilled water) for 4 min with or without test substances. Somata of labelled Müller cells were imaged using confocal microscopy (LSM 510 Meta, Zeiss, Oberkochen, Germany) and their cross‐sectional areas were measured (Zeiss LSM Image Examiner Version 3.2.0.70). In dnSNARE mice, EGFP+ and EGFP‐ cells were imaged and analyzed separately (excitation: 488 nm; emission: 505 nm long‐pass filter).

### 2.8 Histological and immunohistochemical staining

Retinae were immersion‐fixed (4% paraformaldehyde for 2 hr), washed with phosphate‐buffered saline (PBS), embedded in PBS containing 3% agarose (w/v) and cut in 70 µm thick sections using a vibratome. Retinal sections were permeabilized (0.3% Triton X‐100 plus 1.0% DMSO in PBS) and blocked (5% normal goat serum with 0.3% Triton X‐100 and 1.0% DMSO in PBS) for 2 hr at room temperature. Primary antibodies were incubated overnight at 4°C. Sections were washed (1% bovine serum albumin in PBS) and incubated with secondary antibodies (2 hr at room temperature). Cell nuclei were labeled with TO‐PRO‐3 (1:1000; Life Technologies). Retinal whole‐mounts were labeled using a similar protocol, except that tissue was permeabilized by higher concentrations of Triton X‐100 and DMSO (0.3% Triton X‐100 plus 1.0% DMSO in PBS) and secondary antibodies were also incubated at 4°C overnight. Control experiments without primary antibodies showed no unspecific labeling except for the goat‐anti‐mouse secondary antibody which labeled blood vessels (not shown). Images were acquired using confocal microscopy (LSM 510 Meta, Zeiss). Cell nuclei were counted in three retinal layers in 100 µm‐wide areas of the central retina close to the optic nerve head (optical slice thickness, 1.5 µm). The TUNEL assay was performed according to the manufacturer's protocol (Roche Molecular Biochemicals). Free‐floating retinal sections were subjected to a brief microwave treatment in citrate buffer (pH 6.0, 0.1 M) to enhance tissue penetration. After several washing steps, sections were permeabilized (10 min in 4% Triton X‐100), incubated in the labeling solution (90 min), counterstained with TO‐PRO‐3 and mounted on glass slides.

### 2.9 Magnetic‐activated cell sorting of retinal cells

Müller cells were enriched as described (Grosche et al., 2016). Briefly, retinae were digested as described above, incubated with DNase I (200 U/ml), and triturated in extracellular solution containing (mM) 135 NaCl, 3 KCl, 2 CaCl_2_, 1 MgCl_2_, 1 Na_2_HPO_4_, 10 HEPES, and 11 glucose adjusted to pH 7.4 with Tris. After centrifugation, cells were resuspended and incubated in extracellular solution containing biotinylated hamster anti‐CD29 (clone Ha2/5, 0.1 mg/ml, BD Biosciences, Heidelberg, Germany) for 15 min at 4°C. Cells were washed in extracellular solution, spun down, resuspended in the presence of anti‐biotin MicroBeads (1: 5; Miltenyi Biotec, Bergisch Gladbach, Germany) and incubated for 10 min at 4°C. After washing, CD29+ Müller cells were separated using large cell columns according to the manufacturer's instructions (Miltenyi Biotec). To select microglial cells in addition to Müller cells, the retinal suspension was incubated prior to Müller cell selection with CD11b‐microbeads (Miltenyi Biotec) for 15 min at 4°C and microglia were depleted using LS‐columns (Miltenyi Biotec). To quantify cell enrichment, 50 µl of each sample were fixed (4% paraformaldehyde), washed in PBS and deposited on a glass slide. Dried samples were encircled with a PAP pen and subjected to nuclear and immunocytochemical staining using an antibody against glutamine synthetase. Müller cells were identified by the presence of glutamine synthetase and their morphology. Their percentage compared with total count of cell nuclei was determined for respective cell populations.

### 2.10 qRT‐PCR

Total RNA was isolated from whole retinae and from enriched cell populations using the RNeasy Micro Kit (Qiagen, Hilden, Germany). A DNase digestion step was included to remove genomic DNA (Roche). First‐strand cDNAs from 50 ng of total RNA were synthesized using the RevertAid H Minus First‐Strand cDNA Synthesis Kit (Fermentas by Thermo Fisher Scientific, Schwerte, Germany). Primers were desgined using the Universal ProbeLibrary Assay Design Center (Roche). The primers to detect dnSNARE expression covered partially the sequence of the dnSNARE domain and the SV40 polyA tail of the expression construct. Transcript levels of candidate genes were measured by qRT‐PCR using cDNA with the TaqMan hPSC Scorecard™ Panel (384 well, ViiA7, Life Technologies, Darmstadt, Germany) according to the company's guidelines and its cloud‐based online analysis software.

### 2.11 Full‐field electroretinography

Mice were dark adapted for at least 12 hr before recordings and anesthetized by subcutaneous injection of ketamine (65 mg/kg) and xylazine (13 mg/kg). Pupils were dilated with tropicamide eyedrops (Mydriaticum Stulln; Pharma Stulln). Silver needle electrodes served as reference (forehead) and ground (tail) and gold wire ring electrodes as active electrodes. Corneregel (Bausch & Lomb, Berlin, Germany) was applied to keep the eye hydrated and to maintain good electrical contact. ERGs were recorded using a Ganzfeld bowl (Ganzfeld QC450 SCX, Roland Consult, Brandenburg, Germany) and an amplifier with a recording unit (RETI‐Port, Roland Consult). ERGs were recorded from both eyes simultaneously, band‐pass filtered (1–300 Hz) and averaged. Single flash scotopic (dark adapted) responses to a series of ten LED‐flash intensities ranging from −3.5 to 1.0 log cd.s/m^2^ with an interstimulus interval of 2 up to 20 s for the highest intensity were recorded. After 10 min of adaptation to a white background illumination (20 cd/m^2^) single flash photopic (light adapted) responses to three Xenon‐flash intensities (1, 2, and 3 log cd.s/m^2^) were recorded. Responses were quantified based on mean waveform peak amplitude and implicit time. All analysis and plotting was carried out with R 3.2.1 (The R Foundation for Statistical Computing) and ggplot2 2.1.0.

### 2.12 Statistics

All data are expressed as mean ± standard error (*SEM*) unless stated otherwise. Statistical analyses were performed using Prism (Graphpad Software, San Diego, CA). Unless stated otherwise the significance was determined by the nonparametric Mann‐Whitney U test.

## 3 RESULTS

### 3.1 Validation of glia‐specific dnSNARE expression and its impact on transmitter release and volume regulation in Müller cells

**Figure 1:**
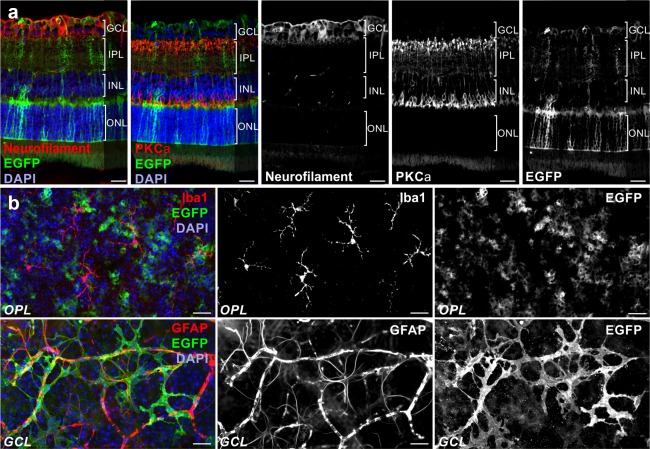
Validation of glia‐specific transgene expression in retinae of dnSNARE mice. Immunohistochemical staining of retinal slices (a) and whole‐mounts (b) demonstrating exclusive EGFP expression as reporter for dnSNARE transgene expression in astrocytes and Müller cells. (a) Micrographs of retinal slices showing EGFP‐positive glial cells, protein kinase Cα (PKCα)‐positive rod bipolar cells and neurofilament‐positive neuronal fibers of the inner retina. Cell nuclei were labeled by DAPI. (b) Micrographs of retinal whole‐mounts showing Iba1‐positive microglial cells and EGFP‐ and GFAP‐positive astrocytes. Note labelling of vessels in the GFAP channel due to unspecific binding of the secondary antibody. GCL = ganglion cell layer; IPL = inner plexiform layer; INL = inner nuclear layer; ONL = outer nuclear layer; OPL = outer plexiform layer. Scale bars, 20 µm [Color figure can be viewed at wileyonlinelibrary.com]

**Figure 2:**
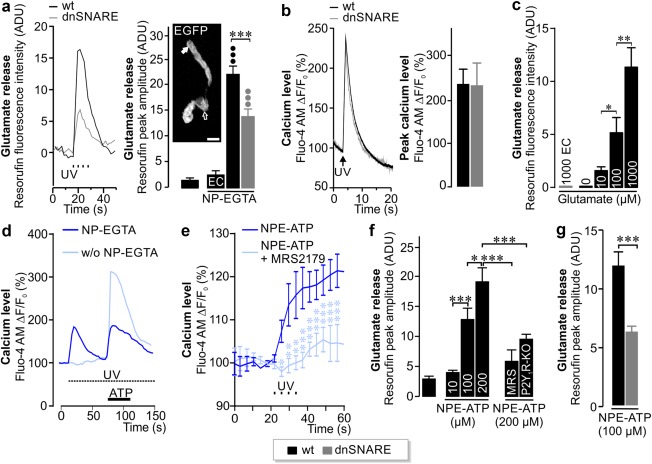
Impact of dnSNARE expression on the glial calcium response and glutamate release. **(**a) *Left*, traces of glutamate release induced by calcium uncaging via four consecutive UV flashes measured above the endfoot of a single Müller cell from a wildtype and a dnSNARE mouse. *Right*, calcium‐induced glutamate release in Müller cells in transgenic compared with wildtype mice. No signal was detected in the absence of NP‐EGTA or glutamate‐specific enzymes (EC = enzyme control) (*n* = 43–55 cells from 4 animals per genotype). *Inset*, EGFP‐positive Müller cell. The open arrow points at the soma, while the filled arrow marks the Müller cell endfoot. Scale bar, 10 µm. Compared with measurements in the absence of NP‐EGTA, but with all enzymes included: •••*p* < .001. (b) Mean microfluorimetric traces (*left*; *n* = 6 cells each) and average sizes (*right*; *n* = 12–16 cells; 2 animals per genotype) of calcium increase in isolated Müller cells from wildtype and dnSNARE mice after a single UV flash uncaging calcium from NP‐EGTA. (c) Enzyme‐ (EC, no glutamate‐specific enzymes) and concentration‐dependent increase of glutamate‐induced resorufin fluorescence indicating the sensitivity of the microfluorometric assay. Bars represent mean ± *SEM* from 7 to 12 measurements from two independent preparations. *p < .05, **p < .01. (d) Traces showing transient increase in intracellular calcium after UV‐induced photolysis in the presence, but not in the absence of NP‐EGTA in acutely isolated Müller cells measured by Fluo‐4 AM (*n* = 6). Control experiments revealed no UV‐induced calcium transients in the absence of NP‐EGTA (*n* = 6) and proved cell viability as ATP (200 µM) induced calcium responses in Müller cells after UV photolysis. (e) Mean calcium concentration in isolated Müller cells before and after UV‐induced uncaging of ATP (100 µM, 4 consecutive UV flashes) in the absence (*n* = 8 cells) or presence of the P2Y_1_ receptor blocker MRS2179 (30 µM, *n* = 7 cells). Compared with measurements in the absence of MRS2179: ***p < .001, **p < .01, *p < .05. (f) Mean size of ATP‐induced glutamate release at different concentrations of NPE‐ATP (*n* = 10 cells from 2 mice), in P2Y_1_R‐KO mice or in the presence of the P2Y_1_ receptor antagonist MRS2500 (MRS, 10 nM) (*n* = 15–20 cells from 2 to 3 animals). (g) Mean ATP‐induced glutamate release (4 consecutive UV flashes) in Müller cells from dnSNARE and wildtype mice (*n* = 57–59 cells from 5 animals). ***p < .001: ADU = arbitrary digital unit [Color figure can be viewed at wileyonlinelibrary.com]

We first examined the expression of the dnSNARE transgene and its impact on Müller cells using different approaches. Immunohistochemical staining of retinal sections and whole‐mounts from transgenic mice revealed expression of EGFP in Müller cells and astrocytes, but not in neurons or microglial cells (Figure 1a,b). We investigated whether dnSNARE expression impacted the calcium‐induced release of the gliotransmitters glutamate and ATP in single acutely isolated Müller cells using enzymatic assays (Slezak et al., 2012). UV photolysis‐induced calcium transients evoked the release of glutamate from Müller cells isolated from wildtype mice after loading with the photolabile chelator NP‐EGTA. In Müller cells from transgenic mice, the release was significantly reduced (Figure 2a), whereas the size and kinetics of UV‐induced calcium transients were similar in cells from both genotypes (Figure 2b) indicating a direct effect of dnSNARE on the release of glutamate. Control experiments confirmed the microfluorimetric detection of glutamate, the absence of NP‐EGTA‐independent release and side effects due to UV exposure (Figure 2a,c,d).

Since photolysis of NP‐EGTA may induce nonphysiological levels of intracellular calcium, we tested next, whether extracellular ATP triggers purinergic receptor‐dependent glutamate release from Müller glia as observed previously in astrocytes (Domercq et al., 2006; Jourdain et al., 2007). Photolysis of caged ATP caused a P2Y_1_ receptor‐dependent release of glutamate from Müller cells: it was attenuated by the P2Y_1_ antagonist MRS2179 (30 µM) and it was reduced in Müller cells isolated from P2Y_1_ KO mice (Figure 2e,f). Müller cells from dnSNARE mice showed reduced P2Y‐dependent glutamate release but calcium transients similar to those from wildtype controls (Figures 2g and 6).

**Figure 3:**
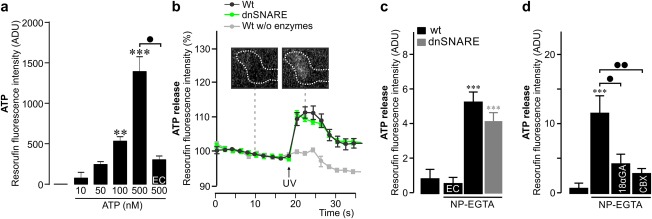
Release of ATP is unaltered in dnSNARE mice. (a) ATP could be detected in a concentration‐dependent manner based on resorufin fluorescence in the modified enzymatic assay as determined by cell‐free measurements on a microplate reader. No or significantly smaller signals were detected in the absence of ATP or ATP‐specific enzymes (EC = enzyme control). Values represent mean ± *SEM* from 4 independent experiments. (b) Increase in ATP release following UV‐induced photolysis of NP‐EGTA in Müller cells from wildtype (Wt, *n* = 7 cells) and dnSNARE mice (*n* = 7 cells). No ATP signal was observed after the removal of the ATP‐specific enzymes. Insets: micrographs showing resorufin fluorescence above a Müller cell endfoot (demarcated by dashed line) before and after the UV pulse. (c) Calcium‐induced ATP release in Müller cells from wildtype (Wt) and dnSNARE mice. No signal was detected in the absence of NP‐EGTA or ATP‐specific enzymes (EC = enzyme control) (*n* = 7–11 cells from 2 animals of each genotype). Compared with measurements in the absence of NP‐EGTA, but with all enzymes included: *** *p* < .001. (d) NP‐EGTA‐induced ATP release in the presence or absence of 18‐α‐glycyrrhetinic acid (18αGA, 50 µM) and CBX (200 µM) (*n* = 10–15 cells). Compared with measurements in the absence of NP‐EGTA: ***p < .001. • *p* < .05, •• *p* < .01 [Color figure can be viewed at wileyonlinelibrary.com]

Previous findings showing SNARE‐dependent ATP release from astrocytes prompted us to establish a modified enzymatic assay to detect ATP release from Müller cells (Figure 3a). UV light induced equal ATP signals in cells from wildtype and dnSNARE mice whereas removal of the respective enzymes prevented the ATP signals (Figure 3b,c). Addition of the connexin hemichannel blockers, 18‐α‐glycyrrhetinic acid (50 µM) and carbenoxolone (CBX) (200 µM) significantly reduced the ATP signals. The data suggest that ATP is released from Müller cells in a SNARE‐independent manner, probably via hemichannels.

**Figure 4:**
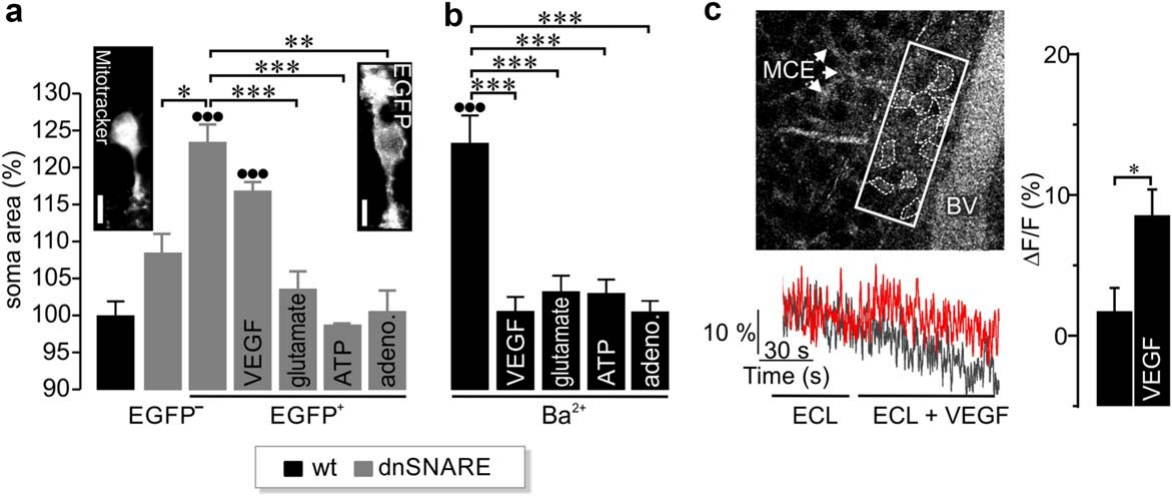
Effect of dnSNARE expression on Müller cell volume regulation. (a, b) The soma area of each cell after 4 min of exposure to hypoosmolar solution was normalized to its size in isoosmolar solution (*n* = 9–15 cells from 2 to 3 mice per genotype). (a) EGFP^‐^ Müller cells showed no increase in soma size whereas EGFP^+^ cells swelled under hypoosmotic conditions. Glutamate (1 mM), ATP (200 µM), and adenosine (10 µM), but not VEGF (10 ng/ml) restored the volume regulation of dnSNARE‐expressing cells. *Insets*, Müller cells from wildtype mice labeled by Mitotracker Orange (10 µM, *left*) and from transgenic mice expressing EGFP (*right*). Scale bars, 5 µm. Compared with measurements of Müller cell soma area in wildtype retinae: •••*p* < .001. (b) Area of Müller cell somata in retinal slices from wildtype mice challenged by hypoosmotic solution in the presence of 1 mM barium. All applied compounds restored volume regulation. Compared with soma area of wildtype Müller cells in the absence of barium ions: •••*p* < .001. (c) *Left, top*, Changes in the Fluo‐4 AM fluorescence were recorded from the area delineated by the rectangle. Dashed lines mark single MCE. BV = blood vessel. *Left, bottom*, representative traces of calcium transients in MCE of retinal whole‐mount preparations from wildtype mice demonstrate VEGF‐induced rises in intracellular calcium (red trace). *Right*, calcium concentration in Müller cells (control: *n* = 52 cells from 2 mice; VEGF: *n* = 81 cells from 8 mice). ***p < .001, **p < .01, *p < .05. Data are represented as mean ± *SEM* [Color figure can be viewed at wileyonlinelibrary.com]

Next, we tested how the dnSNARE transgene affected volume regulation in Müller cells. Previous studies revealed that this process relies on a VEGF‐triggered signaling pathway comprising calcium‐dependent glutamate release, metabotropic glutamate receptors and the nonvesicular release of ATP and adenosine (Brückner et al., 2012; Slezak et al., 2012; Wurm, Pannicke, Wiedemann, Reichenbach, & Bringmann, 2008). As shown in Figure 4a, superfusion with hypoosmotic solution increased the soma size of Müller cells expressing EGFP and dnSNARE, but not of cells from wildtype mice or of EGFP‐negative cells from transgenic mice. The size change in EGFP‐positive cells was abolished by glutamate, ATP and adenosine, but not by VEGF as expected because the latter acts upstream of the glutamate release. Control experiments on Müller cells from wildtype mice confirmed that these compounds compensated the volume increase in hypoosmotic solution induced by barium‐block of inwardly rectifying potassium channels (Figure 4b). Microfluorimetry of retinal whole‐mounts loaded with the calcium indicator Fluo‐4 AM revealed that VEGF enhances the calcium concentration in Müller cells. VEGF was used at the same concentration that prevented barium‐induced swelling in wildtype Müller cells by inducing glutamate release (Figure 4c).

Taken together, these results validated that dnSNARE expression in the retina was specific to Müller cells, that it inhibited a fraction of their calcium‐dependent glutamate release but not of their calcium‐dependent ATP release, and that it impaired their volume regulation.

### 3.2 Ischemia‐induced changes in retinal Müller cells

**Figure 5:**
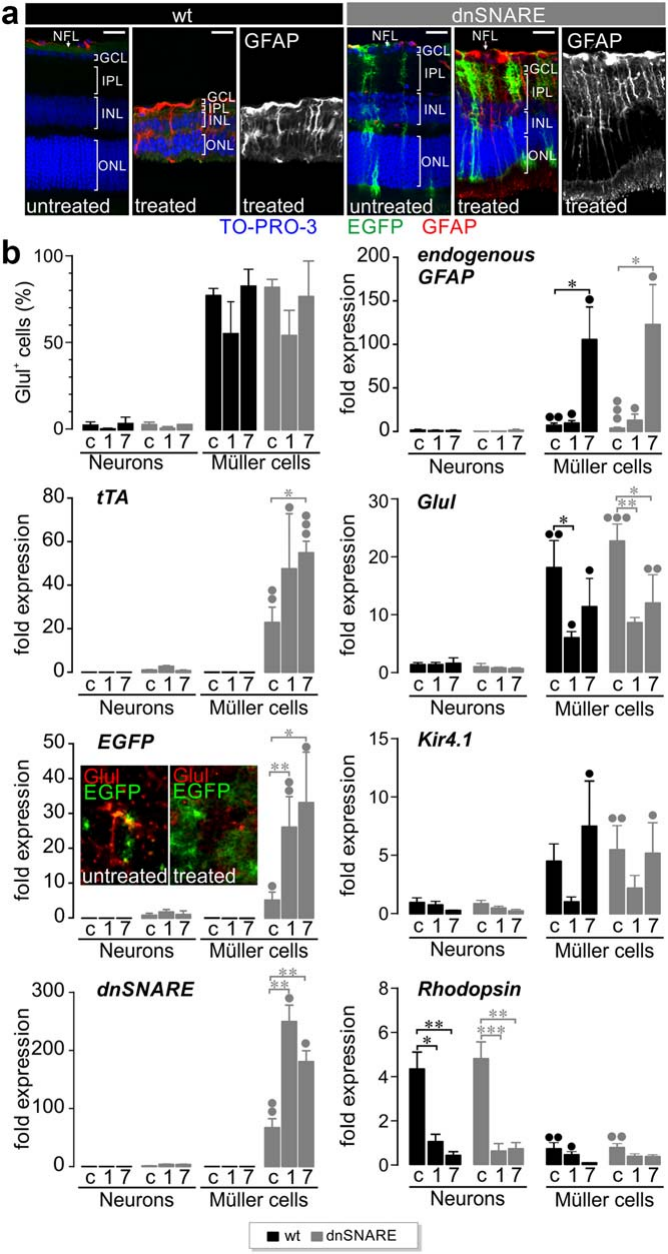
Ischemia‐induced changes in Müller cells. (a) Immunohistochemical (GFAP) and nuclear staining (TO‐PRO‐3) of retinal slices from dnSNARE and wildtype mice 7 (d) days after ischemia/reperfusion. Scale bar, 20 µm. (b) *Top left*, Mean percentage of glutamine‐synthetase (Glul) positive cells in retinal cell suspensions from untreated (c) and postischemic (1 d (1), 7 d (7); *n* = 2–3 independent experiments) retinae of wildtype and transgenic mice that were subjected to magnetic‐activated cell sorting‐based cell enrichment for Müller cells. Neuronal fractions (neurons) were depleted from Müller cells. Other graphs in the panel show the increase of indicated transcript levels in enriched Müller cells compared with the expression in the neuronal fraction from the control eye. Note absence of glial and neuronal markers from neuronal and glial preparations, respectively, the postischemic loss of rhodopsin‐positive photoreceptors and the upregulation of GFAP‐dependent transgenes. °°°*p* < .001; °°*p* < .01; °*p* < .05: significant differences in transcript expression in Müller cells compared with the neuronal fraction from the same retinal condition. Values were obtained from 4 to 6 independent experiments. Expression levels for rhodopsin were normalized to the expression level in Müller cells isolated from untreated control retinae. All other genes expression levels were normalized to the expression level in the neuronal fraction from dnSNARE mice of untreated control eyes. ***p < .001, **p < .01, *p < .05. Inset shows micrographs of retinal whole‐mounts from a dnSNARE mice with a considerably larger area of tissue being covered by EGFP‐positive (green) Müller cell processes whereas glutamine synthetase (red) labeling is reduced and diffuse in the postischemic retina. Scale bar, 20 µm [Color figure can be viewed at wileyonlinelibrary.com]

To determine whether and how calcium‐dependent release from Müller cells contributes to pathologic changes ensuing transient ischemia, we subjected transgenic and wildtype mice to an ischemia/reperfusion model, where retinal blood flow is blocked for 90 min by experimentally raised intra‐ocular pressure (Pannicke et al., 2014). As a first step, we examined how Müller cells react to transient ischemia. Seven days after transient ischemia, dnSNARE mice showed a strongly increased density of EGFP‐positive Müller cells (71 ± 6% of all Müller cells; *n* = 6 animals) compared with nonoperated dnSNARE mice (43 ± 7%; *n* = 6 animals). This change was probably caused by ischemia‐induced activation of the GFAP promoter. Indeed, immunohistochemical staining revealed a strong postischemic increase of GFAP expression in retinae from both wildtype and dnSNARE mice (Figure 5a). This was further confirmed by PCR analysis of selected transcripts in enriched glial and neuronal cell preparations from retinae of wildtype and transgenic mice. The glial population, which consisted mainly of glutamine‐synthetase positive Müller cells (Figure 5b), showed strongly increased levels of the tTA (tetracycline transactivator), EGFP and SNARE transgene, whose expression is controled by the human GFAP promoter (Figure 5a). Notably, these transgenes were absent from retinal neurons isolated from transgenic mice confirming their glia‐specific expression. We observed 67 (± 15.5)‐fold higher levels of dnSNARE transcripts in Müller cells compared with neurons, whereas the well‐established Müller cell marker glutamine synthetase or Kir4.1 were only enriched by 18.1 (± 4.8)‐fold or 4.8 (± 1.7)‐fold, respectively, demonstrating robust transgene‐expression in retinal Müller cells (Figure 5b). Ischemia‐induced activation of the GFAP promoter was further confirmed by a parallel increase of endogenous GFAP transcripts in transgenic and wildtype mice. The levels of glutamine synthetase and Kir4.1 were only transiently decreased. Interestingly, rhodopsin transcripts were expressed in the neuronal population and strongly decreased by ischemia indicating degeneration of photoreceptors (Figure 5b).

**Figure 6:**
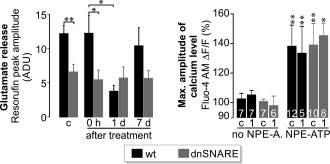
Altered glutamate release from Müller cells of the postischemic retina. *Left*, mean sizes of ATP‐induced glutamate release (4 consecutive UV flashes) in single Müller cells from transgenic and wildtype (wt) mice at the indicated conditions (c: untreated; 0 hr: after 90 min ischemia, 1 and 7 d postischemia) (*n* = 15–44 cells from 3 to 4 animals per group). **p < .01; *p < .05. ADU = arbitrary digital units. *Right*, mean amplitudes of ATP‐induced calcium responses (normalized to UV‐flash induced changes in the absence of NPE‐ATP) in Müller cells isolated from untreated control eyes (c) or one day after transient ischemia (1) from mice with indicated genotypes (*n* = 5–12 cells from 2 mice per group). **p < .01; *p < .05

Next, we analyzed whether and how ischemia affected the ATP‐induced glutamate release from Müller cells. In wildtype animals, the glial glutamate release was only transiently reduced one day after the insult and returned to a normal level at 7 d (Figure 6). In mice bearing the dnSNARE transgene, glial ATP‐induced glutamate release was constantly lower compared with wildtype mice regardless of ischemia (Figure 6) except at 1 d after ischemia. The ischemic insult did not affect the ATP‐induced calcium response in Müller cells from wildtype or transgenic mice (Figure 6).

### 3.3 Inhibition of glial SNARE‐dependent release reduces postischemic loss of neurons

**Figure 7:**
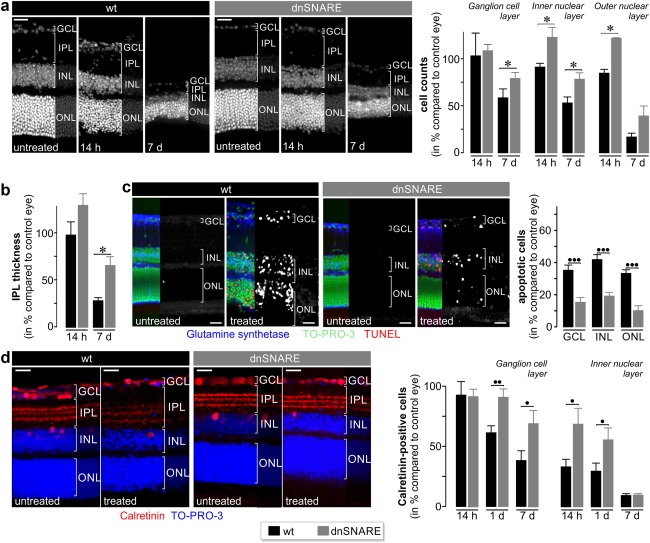
Reduction of neuronal cell loss by glial dnSNARE expression. (a) *Right*, Mean counts of DAPI‐positive cell nuclei (*left*, see exemplary micrographs) in the indicated retinal layers at 14 hr and 7 (d) days after transient ischemia. (b) Mean thickness of the IPL at 14 hr and 7 d postsurgery. Cell number (a) and thickness (b) were normalized to values from the retina of the untreated contralateral eye (*n* = 4–5 slices from 3 to 11 animals per genotype and time point). *p < .05. (c) *Left*, Retinal sections subjected to TUNEL, nuclear (TO‐PRO‐3) and glutamine synthetase staining to reveal Müller cells. Grayscale micrographs show the TUNEL‐signal. *Right*, mean counts of TUNEL‐positive cells at 1 d after transient ischemia normalized to total number of nuclei in the respective retinal layer (4–5 central retinal slices per animal; 5 animals per genotype). •••*p* < .001. (d) *Left*, Retinal sections subjected to nuclear (TO‐PRO3) and calretinin staining—a marker of amacrine cells in the INL and of ganglion cells and displaced amacrine cells in the GCL 1 d postsurgery. Note better preservation of calretinin‐positive dendrites extending into the IPL in postischemic retinae of dnSNARE mice. *Right*, mean counts of calretinin‐positive cells in the GCL and INL of retinal sections prepared 14 hr, 1 d, and 7 d postsurgery (4–5 central slices per animal, 3–11 animals per genotype). Values were normalized to the number of cells found in the respective nonoperated contralateral eyes. ••*p* < .01; •*p* < .05. ONL = outer nuclear layer [Color figure can be viewed at wileyonlinelibrary.com]

Next, we studied how dnSNARE expression influenced pathologic changes in postischemic retinae. A cardinal feature of the ischemia/reperfusion model is the robust loss of neurons as shown by the reduction of rhodopsin (Figure 5b) and the thinning of retinal layers (Figure 7a,b). While all morphological parameters (e.g., cell counts and IPL thickness) investigated were not significantly different in untreated retinae of wild type and dnSNARE mice (data not shown), our quantitative analysis revealed that dnSNARE expression reduced the ischemia‐induced loss of cells in the nuclear layers and diminished the thinning of the inner plexiform layer (IPL) at different time points after surgery compared with wildtype animals (Figure 7a,b). These findings were confirmed by TUNEL staining of apoptotic cells. One day after surgery, when the neuronal cell loss peaks (Kuroiwa et al., 1998), retinae from dnSNARE mice showed significantly less apoptotic cells accross all layers compared with those from wildtype littermates (Figure 7c). Since retinal ganglion cells and amacrine cells are particularly susceptible to postischemic hyperexcitation (Neufeld et al., 2002; Pannicke et al., 2014), we analyzed their density using immunohistochemical staining for the specific marker calretinin (Figure 7d). Quantitative analysis revealed a reduced loss of calretinin‐expressing cells in the GCL 1 and 7 d after surgery in dnSNARE mice compared with wildtype controls (Figure 7d). The loss of calretinin‐positive cells in the INL was also reduced except for the 7 d time point. Together, these results indicated that expression of dnSNARE in Müller cells reduces the loss of neurons in specific retinal layers.

**Figure 8:**
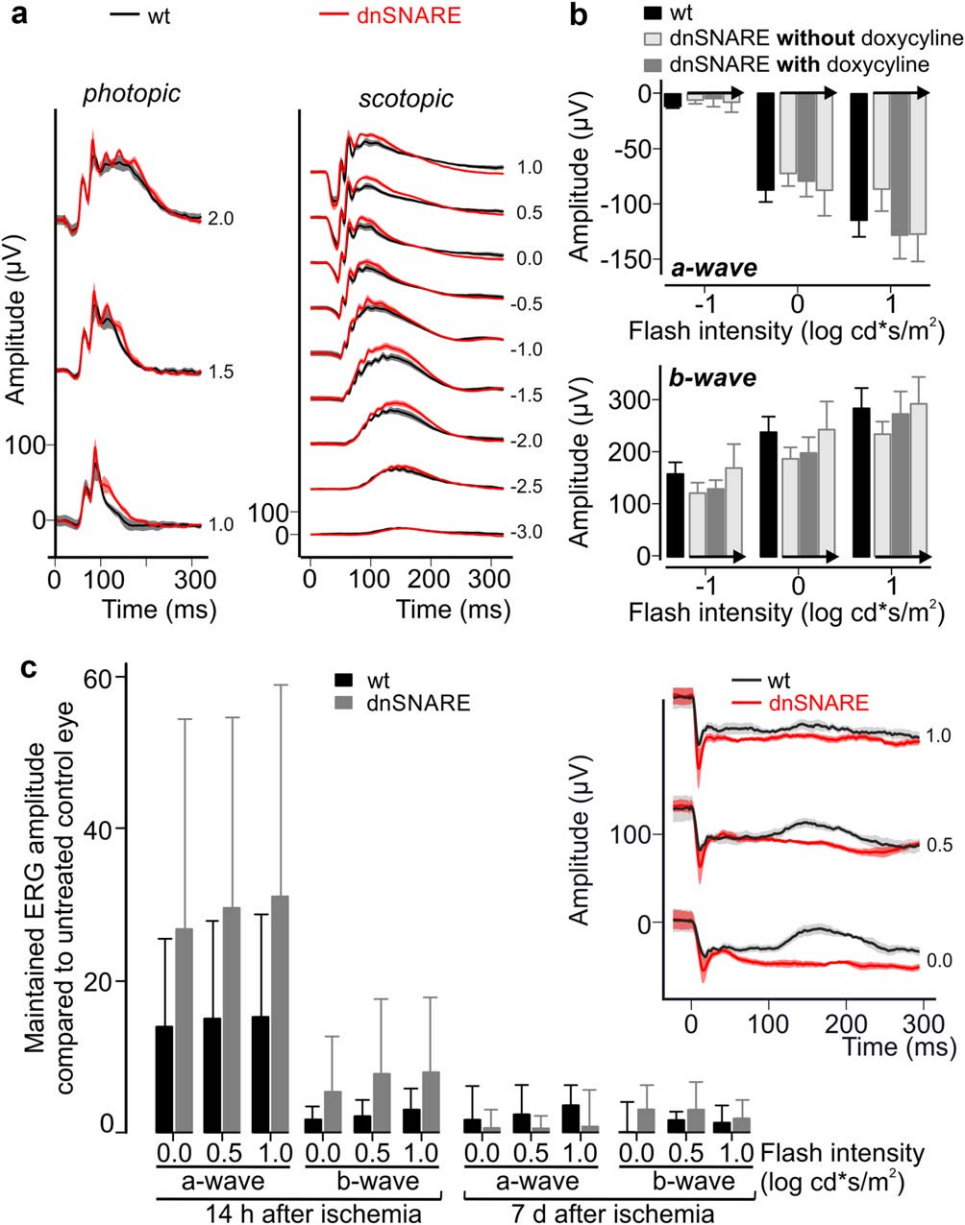
Full‐field ERGs from wildtype and dnSNARE mice. (a) Single flash ERG recordings from adult wildtype and dnSNARE mice (*n* = 8 mice per genotype) under indicated conditions. Flash intensities are given as small numbers to the right of each trace and in (log (cd*s/m^2^)). (b) Maximal amplitudes of scotopic light responses from dark‐adapted untreated wildtype and dnSNARE mice (*n* = 4 mice per genotype). Light responses of the same dnSNARE mice were measured at three consecutive time points (the arrow indicates direction of the time line): before doxycycline administration (open bar), after two weeks of continuous doxycycline treatment (filled bar, via the drinking water) and after wash‐out of doxycycline (open bar again, normal drinking water for three weeks). (c) Mean amplitudes of a‐ and b‐waves recorded 14 hr and 7 d after transient ischemia in wildtype and dnSNARE mice. Values were normalized to maximal amplitudes recorded from untreated contralateral eyes of each mouse (*n* = 6 mice per genotype) and are given with standard deviations. *Inset*, traces of light responses to scotopic stimulation from eyes of wildtype and dnSNARE mice 14 hr after surgery. Flash intensities are given as small numbers to the right of each trace and are given in [log (cd*s/m^2^)] [Color figure can be viewed at wileyonlinelibrary.com]

To explore whether and how the dnSNARE transgene affected the functional integrity of the postischemic retina, we recorded electroretinograms (ERGs) (Figure 8). Control experiments confirmed that expression of the dnSNARE transgene in Müller cells or its transient suppression by administration of doxycycline did not affect ERG parameters (Figure 8a,b) confirming results obtained with inducible expression of Botulinum neurotoxin B (iBot mice; Slezak et al., 2012). Transient ischemia strongly reduced the a‐ and b‐wave in wildtype mice already 14 hr after surgery, and this effect was much less pronounced in dnSNARE mice retina (Figure 8c) indicating that dnSNARE expression dampens the pathologic impairment of retinal light responses. However, at 7 d after surgery, the visual responses disappeared in wildtype and transgenic mice probably due to the loss of photoreceptors, which could not be prevented by glial dnSNARE expression.

## 4 DISCUSSION

In this study, we used a transgenic mouse model to investigate the impact of glial SNARE‐dependent exocytosis on postischemic neurodegeneration in the retina in vivo. We show that glia‐specific inhibition of SNARE‐dependent signaling by dnSNARE expression reduces exocytotic release of glutamate from Müller cells, impairs glial volume regulation and protects retinal neurons from postischemic dysfunction and degeneration, whereas ATP release by Müller cells remained unaltered.

### 4.1 Glia‐specific dnSNARE expression inhibits exocytotic transmitter release and impairs volume regulation

Our findings validate dnSNARE mice as a tool to interfere with SNARE‐dependent processes in retinal glial cells. Immunohistochemical staining of retinal sections (Figure 1) and transcript profiling revealed that the transgene is exclusively present in glial cells (Figure 5b). The human GFAP promoter fragment driving transgene expression faithfully recapitulated the activity of the endogenous GFAP promoter. Notably, both promoters were activated by ischemia and caused a parallel increase of transcripts encoding GFAP and dnSNARE/EGFP (Figure 5b). Neuronal transgene expression previously reported in cortex and hippocampus of dnSNARE mice (Fujita et al., 2014) was not detectable in the retina indicating regional differences in the targeting efficacy of transgenic lines (Pfrieger & Slezak, 2012).

Expression of the dnSNARE transgene reduced the amount of glutamate release from Müller cells regardless of its induction by calcium (36% less release) or by ATP (48% reduction) (Figure 2a,f). These results are similar to what was observed previously after glial expression of Botulinum neurotoxin serotype B (38% less) or pharmacological blockade of vesicle loading with glutamate by application of bafilomycin A1 (reduced by 31%; Slezak et al., 2012). In the end, a very consistent reduction of glutamate release was measured in the three independent experimental settings. This suggests that all approaches (a) were similarly effective (quite likely close to 100% given the high consistency of the results), (b) could hence serve as valuable tools to analyze the impact of an absent glial exocytotic release. Moreover, they corroborate the presence of glial glutamate release that is SNARE‐independent and mediated by alternative pathways (Hamilton & Attwell, 2010; Montero & Orellana, 2015; Woo et al., 2012).

In concordance with Slezak et al. (2012), we also found that interference with SNARE‐dependent signaling impaired the ability of Müller cells to maintain their volume when challenged by hypotonic solution (Figure 4). Glutamate has been demonstrated to evoke release of ATP from Müller cells within the volume regulatory signaling cascade (Wurm et al., 2008). Therefore, the block of swelling of EGFP‐positive cells by glutamate (Figure 4a) suggests that the release of ATP is not affected in dnSNARE mice. This is in agreement with our direct measurements of ATP release by single Müller cells (Figure 3). The defect in volume regulation was absent in Müller cells that did not express the dnSNARE and the EGFP transgenes. This particular pool of cells may prevent major morphological or functional alterations in retinae from transgenic mice. In particular, their presence may explain why light‐evoked responses in ERGs appear fairly normal in dnSNARE mice as well as in iBot mice (Slezak et al., 2012) compared with wildtype animals. Taken together, two independent genetic approaches to inhibit SNARE‐dependent processes in glial cells provide converging results, a partial inhibition of calcium‐dependent glutamate release from individual cells and impaired volume regulation.

### 4.2 Contribution of SNARE‐dependent processes to postischemic neuronal dysfunction and degeneration

Using transcript analyses, ERGs and histochemical staining, we show that transient ischemia/reperfusion impaired light‐evoked responses in the retina as early as 14 hr after surgery and caused massive degeneration of photoreceptors and other types of retinal neurons within the following days. Expression of dnSNARE in glial cells markedly reduced the early defects in light‐evoked responses of photoreceptors (a‐wave) and inner retinal cell types (b‐wave) following ischemia. Obviously, the functional integrity of retinal cells is highly sensitive to ischemic damage and impaired long before cell loss. Our observation that transgene expression protected neurons in the inner retina but not photoreceptors underline previous observations that among the retinal neurons, ganglion and amacrine cells are most susceptible to glutamate‐induced hyperexcitation in the adult stage (Adachi et al., 1998; Lam, Abler, & Tso, 1999). Under normal conditions, Müller cells take up extracellular glutamate by dedicated transporters such as the glutamate‐aspartate transporter (Bringmann et al., 2013; Rauen, 2000), which depend on a negative membrane potential of Müller glia (Brew & Attwell, 1987; Bringmann et al., 2013; Sarantis & Attwell, 1990). Depolarization of Müller cell membranes by high extracellular potassium concentrations in ischemic tissue impairs glutamate uptake (Billups & Attwell, 1996; Maguire et al., 1998; Szatkowski, Barbour, & Attwell, 1990) ultimately increasing extracellular glutamate levels. Cells highly susceptible to glutamate‐induced neurotoxicity die and thereby release large amounts of ATP, which in turn aggravates the disastrous rise of extracellular glutamate by triggering its release from Müller glia. This process is probably interrupted by reduced glutamate release from Müller cells expressing the dnSNARE transgene. It has to be considered that release of glutamate from glial cells is small compared with release from presynaptic terminals. This is probably one of the reasons why blockade of SNARE‐dependent processes in glial cells cannot prevent ultimate neuronal death in our ischemic model. Nevertheless, we observe a transient amelioration in neuronal function and a delayed loss of specific cells in dnSNARE mice revealing a contribution of SNARE‐dependent processes in glial cells to postischemic neuronal dysfunction and degeneration. At present, we cannot exclude that SNARE‐dependent processes in glial cells other than glutamate release have contributed to reduced neurodegeneration. We have demonstrated that ATP release by Müller cells is unaltered by dnSNARE expression, however, a potential release of other gliotransmitters, for example, D‐serine, remains to be investigated.

Taken together our results reveal that dnSNARE expression in Müller cells protects retinal function in the early phase of ischemia and reduces the loss of neurons in the inner retina although it remains to be demonstrated whether there is a direct causal relationship between glial transmitter exocytosis and neurodegeneration. These findings emphasize the need to further investigate the role of SNARE‐dependent processes in glial cells under normal and pathological conditions and to explore their potential as therapeutic targets.

## CONFLICT OF INTEREST

The authors declare no competing financial interests.
